# Assistive Robotic Arm to Support Activities of Daily Living in Individuals With Tetraplegia: Protocol for a Real-World Convergent Parallel Mixed Methods Feasibility Study

**DOI:** 10.2196/78339

**Published:** 2026-03-03

**Authors:** Vera Fosbrooke, Aline Christen, Barbara Catherine Wortmann, Iris Theodora Maria de Boer, Raphael Rätz, Julian Frederik Rösch, Gabriel Gruener, Anja M Raab

**Affiliations:** 1 Department of Health, School of Health Professions, Bern University of Applied Sciences, Murtenstrasse 10, Bern, 3012, Switzerland, 41 31 848 37 2; 2 Department of Engineering and Information Technology, School of Engineering and Computer Science, Bern University of Applied Sciences, Biel, Switzerland

**Keywords:** WMRAs, robotic arm, tetraplegia, usability, health economic analysis, wheelchair-mounted robotic arms

## Abstract

**Background:**

Tetraplegia, often resulting from cervical spinal cord injury, may lead to significant motor and sensory loss, severely impacting independence and quality of life (QoL). Assistive technologies, such as wheelchair-mounted robotic arms (WMRAs), offer potential to enhance autonomy in daily living. However, adoption remains limited due to high costs, complex controls, and insufficient end user involvement. Robust evidence on their real-world effectiveness, particularly post hospitalization, is still lacking.

**Objective:**

This study aims to explore the feasibility, usability, and user experience of a WMRA for individuals with tetraplegia in real-life posthospitalization settings. It aims to evaluate its support in activities of daily living and conduct a preliminary health economic analysis comparing cost-effectiveness and QoL outcomes with standard care.

**Methods:**

This mixed methods feasibility study will be conducted in posthospitalization settings in Switzerland. Up to 15 participants with upper limb impairments (SCI C0–Th1, AIS A–D) using powered wheelchairs will be recruited. They will use the robotic arm for 6 consecutive days. An equal number of participants will be recruited for the economic analysis group. Quantitative data will be collected at baseline and postintervention via standardized questionnaires (Post-Study System Usability Questionnaire, National Aeronautics and Space Administration Task Load Index, European Quality of Life 5-Dimension 5-Level questionnaire, Visual Analogue Scale, adapted version of the Canadian Occupational Performance Measure, and Client Socio-Demographic and Service Receipt Inventory-European Version), while qualitative feedback will be gathered through an informal questionnaire and semistructured interviews. Feasibility will be assessed through task performance and health economic analysis. The latter will include quality-adjusted life years, which quantify quality and length of life, and modeling the Incremental Cost-Effectiveness Ratio, which compares the cost-effectiveness of the intervention based on cost per quality-adjusted life years gained. Findings will be integrated in line with the convergent parallel mixed methods design.

**Results:**

Recruitment began in April 2025 and is ongoing as planned; full feasibility, usability, and economic results will be reported upon study completion. We expect the robotic system to reduce caregiver time and associated costs, while enhancing autonomy, QoL, and mental well-being. Potential technical and recruitment challenges have been identified and mitigation strategies planned. Evaluating real-life use of a WMRAs, this study could support the wider adoption of assistive robotic technologies.

**Conclusions:**

This research offers key insights into the feasibility, usability, and economic value of robotic assistance for individuals with tetraplegia and will help inform future development and scale-up studies.

## Introduction

Tetraplegia is a severe form of spinal cord injury (SCI) resulting from damage to the cervical spine, leading to partial or complete loss of motor and/or sensory function in all 4 extremities and the trunk [1,2]. Globally, SCI affects approximately 15.4 million persons [3], with an annual incidence of 250,000 to 500,000 new cases according to the World Health Organization [2]. Individuals with tetraplegia face extensive challenges in daily life, including limitations in mobility, personal care, and social participation [2,4]. These restrictions significantly reduce autonomy and quality of life (QoL), while also placing a substantial economic burden on both affected individuals and health care systems. According to Pacheco Barzallo et al [5], it has been estimated that persons with SCI in Switzerland use health care services 11 times more (including physiotherapists, nursing services, general practitioners, and specialists) than the healthy population and 4 times more than persons with other chronic health conditions. Moreover, caregivers, especially family members providing unpaid care, often experience heightened psychological stress and are at increased risk of developing mental health conditions [4,6].

Assistive technologies (ATs) play a crucial role in mitigating the effects of physical impairments by enhancing independence and enabling participation in activities of daily living (ADLs) [3,7,8]. AT encompasses a wide range of tools, from adaptive cutlery to advanced robotic systems. Individuals with tetraplegia particularly benefit from wheelchair-mounted robotic arms (WMRAs), which enhance care and promote independence across different areas [9,10]. Examples include the Functional Robot with Dexterous Arm and User-Friendly Interface for Disabled People (FRIEND) system, a wheelchair-mounted robotic manipulator designed to assist users with tetraplegia in tasks, such as drinking and eating [11], or the Jaco 2 robotic arm (Kinova Inc), which has been applied in various contexts, such as adaptive feeding systems [12]. However, their adoption into daily life remains limited. Most devices are still in research or prototype phases, tested primarily in controlled experimental settings with participants without disabilities [7]. The high costs, lack of personalization, and the need for end users to be heavily involved in the development process contribute to the low acceptance and small market transfer of these devices [3,4,13]. The 3 robotic arm models that have reached the commercial market (the Exxomove Bateo, the iARM, and the JACO robotic arm) all lack robust scientific evidence demonstrating their long-term efficacy for individuals with tetraplegia [7,14,15]; furthermore, most published research focuses on technical feasibility only [4].

The current state of research, along with the lack of high-quality studies evaluating practical effectiveness of assistive robotic systems for individuals with tetraplegia in posthospitalization settings, led us to the following objectives of our study:

1. Evaluate the feasibility of a WMRA in supporting ADLs for individuals with tetraplegia.
2. Assess user satisfaction, usability, and perceived autonomy in ADLs involving the robotic arm.
3. Collect qualitative and quantitative data using a mixed methods approach to inform further development of user-centered robotic AT systems.
4. Conduct a health economic analysis to assess the cost-effectiveness of the robotic arm in everyday use compared to formal and informal care, considering both direct and indirect costs (eg, care time and productivity loss), and linking these to outcomes (health-related QoL and perceived independence).

## Methods

### Design and Setting

This study uses a mixed methods research design to evaluate the feasibility and usability of a WMRA for individuals with tetraplegia. The convergent parallel design facilitates a concurrent collection of both quantitative data from standardized and nonstandardized questionnaires and qualitative insights from individual semistructured interviews, allowing for a holistic understanding of system performance and user experiences [16]. The parallel data collection enables an alignment and integration of the findings as well as the use of traditional analysis methods of quantitative and qualitative data, relying on familiar, well-established techniques.

This study is conducted in a range of posthospitalization care environments including inpatient rehabilitation facilities, home settings (with or without support by informal caregivers and/or community-based services), and nursing homes or long-term care facilities in Switzerland. This aims at maximizing external validity and informing implementation across different care pathways, reflecting on real-world contexts in which the WMRA will be used.

The robotic arm system evaluated in this study is a prototype developed by the Bern University of Applied Sciences in Switzerland using a user-centered approach. The system is built around a Kinova Gen3 robotic arm with 7 degrees of freedom [17] equipped with a customized Robotiq 2 F-85 gripper (Robotiq) [18] and is designed to be mounted on a powered wheelchair. A 3D camera located near the gripper provides visual input, which is processed by machine learning algorithms to identify and localize objects. This enables the robotic arm to approach and grasp items semiautonomously, thereby improving efficiency in task execution. A graphical user interface (GUI) displayed as a webpage on a tablet near the user offers both live and processed camera feeds and control of the robot. The user can interact with the GUI in the same way a mobile phone can be controlled in everyday life, either via the touch input on the tablet’s touch screen, via voice commands (Android Voice Access) [19] or through the wheelchair’s joystick (eg, joystick or chin joystick). The robotic arm system was designed with a focus on ease of use and intelligent functionality. Importantly, its software architecture is hardware-independent, offering flexibility in implementation. The fact that the robotic system is still a prototype offers a significant advantage, enabling the incorporation of user feedback to make necessary adjustments and continuous improvements, supporting an iterative design approach.

### Reporting Guidelines

The Good Reporting of a Mixed Methods Study (GRAMMS) is used as a reporting guideline to improve the quality and transparency of this mixed methods research [20]. The evaluated items of the GRAMMS checklist at this time point of the research are provided in Checklist 1.

### Participants and Recruitment

Participants eligible for this study are adults (≥18 y) and live in a posthospitalization care environment (inpatient rehabilitation facilities, home settings with or without support by informal caregivers and/or community-based services, and nursing homes or long-term care facilities) in Switzerland. They must have an upper limb impairment due to a complete or incomplete cervical SCI (neurological levels C0 to Th1), classified according to the American Spinal Injury Association (ASIA) Impairment Scale (AIS) as grades A to D [21]. All participants are required to use a powered wheelchair, and their experience in using a WMRA is not considered as an exclusion criterion. They need to be able to understand and communicate in German, French, Italian, or English. Individuals are excluded from participation in this study if they are unable to operate AT for the upper extremities due to psychological or cognitive limitations. Further exclusion criteria include the presence of a progressive secondary condition that is expected to further impair upper limb function, a diagnosis of a functional neurological disorder, or current pregnancy.

Purposeful sampling [16] using a snowball approach is applied for recruitment, where study team members contact possible participants within their individual network and ask for interest in the study by phone or via email. A flyer containing information about the study and the recruitment is shared on social media (LinkedIn [Microsoft Corp], Facebook [Meta], and other websites) as well as with institutions connecting potential participants. Prospective participants receive detailed information about the study’s purpose and their rights through an informed consent form, which can be signed by the participant or a legal representative. Participants are informed that participation is voluntary, that they may withdraw consent at any time without affecting their subsequent medical care or treatment, and that no compensation will be provided for participation in the study.

### Study Endpoints

The main primary endpoint of this study is to assess the feasibility of using the robotic arm in supporting individuals with tetraplegia performing ADLs in a posthospitalization setting. Feasibility is evaluated through 3 predefined tasks: pickup, drink, and push a button, which serve to assess the usability and effectiveness of the robotic arm. A visual outline of the assessment of the 3 tasks is provided in the Multimedia Appendix 1. The robotic arm will be considered feasible if participants demonstrate the ability to complete at least 2 of the 3 predefined ADL tasks (pickup, drink, and push a button) independently in ≥70% of attempts, achieve a usability score equivalent to a System Usability Scale ≥68 (indicating good usability) [22], and report no excessive cognitive or physical workload as measured by the National Aeronautics and Space Administration-Task Load Index (NASA-TLX). Meeting these criteria collectively is considered an indication that the robotic arm is functionally effective, usable, reliable, and acceptable for supporting ADLs in a real-world setting.

The user-friendliness of the system is measured using the Post-Study System Usability Questionnaire (PSSUQ), a standardized 19-item instrument [23]. The NASA-TLX assesses the perceived cognitive and physical workload of the participants associated with controlling the robot arm when performing ADLs [24]. Additionally, the qualitative data collection aims to gain deeper insights into the physical relief in everyday life and the user experiences. For this, an informal questionnaire developed by the research team evaluates the feasibility, usability, and real-world impact of the robotic arm after use. Based on a semistructured guide, individual feedback is collected from every participant in an interview conducted via smartphone or online via Microsoft Teams. The semistructured interview guide was developed by a member of the research team (ITMdB) and has been reviewed by 2 physiotherapists (AMR and BW), 3 engineers (AC, RR, and JFR), and 1 anonymous individual with tetraplegia to ensure comprehensibility and relevance, and to improve its validity. An outline of the semistructured interview guide is provided in the Multimedia Appendix 2.

Secondary study endpoints include the QoL of the participants assessed using the EQ-5D-5L [25], as well as pain in the upper extremities determining the localization and quality, quantified using a Visual Analogue Scale (VAS) [26]. Self-perceived performance and satisfaction in ADLs are evaluated using an adapted version of the Canadian Occupational Performance Measure (aCOPM) [27], modified to focus on the 10 most important ADLs for individuals with tetraplegia as identified by Hutmacher et al [28]. Information on the sociodemographic background and the use of health and social care services by the study participants are collected using the Client Socio-Demographic and Service Receipt Inventory-European Version (CSSRI-EU) [29]. The inventory captures the type and amount of informal and formal care received (eg, h per wk), out-of-pocket expenses for assistive devices or services, perceived financial burden due to disability-related costs and employment status. The CSSRI-EU has been translated into German and adapted to the structure of the Swiss health and social care system by experts, including relevant terminology, service categories, and financing structures. In addition, it was extended by study-specific items to capture relevant health economic data eg, productivity losses of informal caregivers by the research team.

Other study endpoints include demographic and personal parameters of the participants (age, sex, address, phone number, and email) as well as medical parameters (time after injury, height of the lesion, completeness of injury, spasticity and tonus of upper extremities, and mobility of upper extremities), and technical parameters about the wheelchair and the living setting (brand and model of powered wheelchair, home setting, limited everyday activities, assistance in everyday life, current aids for performing everyday activities).

### Participation Timeline

At least 10 days prior to the clinical phase, participants will receive detailed study information and an informed consent form via email. The signed consent form will be collected at least 7 days before the clinical phase. Upon the receipt of the informed consent form, the questionnaire on demographics, EQ-5D-5L, pain with the VAS, aCOPM, and the CSSRI-EU will be handed out to the participants via email.

All assessments will take place at the posthospital setting where the patient resides. First, baseline assessment of the 3 tasks (pickup, drink, and push a button) will be collected. The robotic arm will then be mounted on the personal wheelchair of the participant by an engineer of the research team. The system will be calibrated, and training is provided based on a strict manual given by the engineer, as well as individual testing of the system by the participant. The training lasts at least 45 minutes and only ends when the participant has no further questions. After training, participants must be able to issue robot commands via at least one supported input pathway (touch, voice, or wheelchair joystick or chin joystick). The robotic arm will then be steered by the participant him or herself and the preassessment of the 3 tasks (pickup, drink, and push a button) performed, as well as questionnaires (PSSUQ and NASA-TLX) filled in. The participant will now be using the robotic arm in ADLs for a total of 6 consecutive days at home. During the clinical test phase, daily log files of the robotic arm will automatically be sent to the responsible researcher through a mobile internet connection. Postassessment of the 3 tasks (pickup, drink, and push a button) as well as postquestionnaires (PSSUQ, NASA-TLX, EQ-5D-5L, as well as the informal questionnaire on the feasibility and usability) will be collected after the 6 consecutive days. The robotic arm will then be removed from the wheelchair by a member of the research team. Two days later, the aCOPM and the VAS will be distributed online to the participants via REDCap (Research Electronic Data Capture; Vanderbilt University). Around 2 to 5 days after the removal of the robot arm, qualitative data, based on the semistructured interview guide, will be collected individually either via telephone or online via Microsoft Teams. The interviews will be held by a member of the research team in German unless Swiss German is preferred and take about 30 minutes.

Underlining the importance of understanding individual variability and following comparable AT research, screening and recruitment will continue until the target sample size of a maximum of 15 study participants using the robotic arm is reached [30-32]. The target sample size follows recommendations of guidelines of comparable research where sample sizes of 30 participants and less are expected to reach saturation in quantitative studies, as well as practical considerations such as a realistically achievable number of participants meeting the inclusion criteria [33,34].

**Figure 1.**
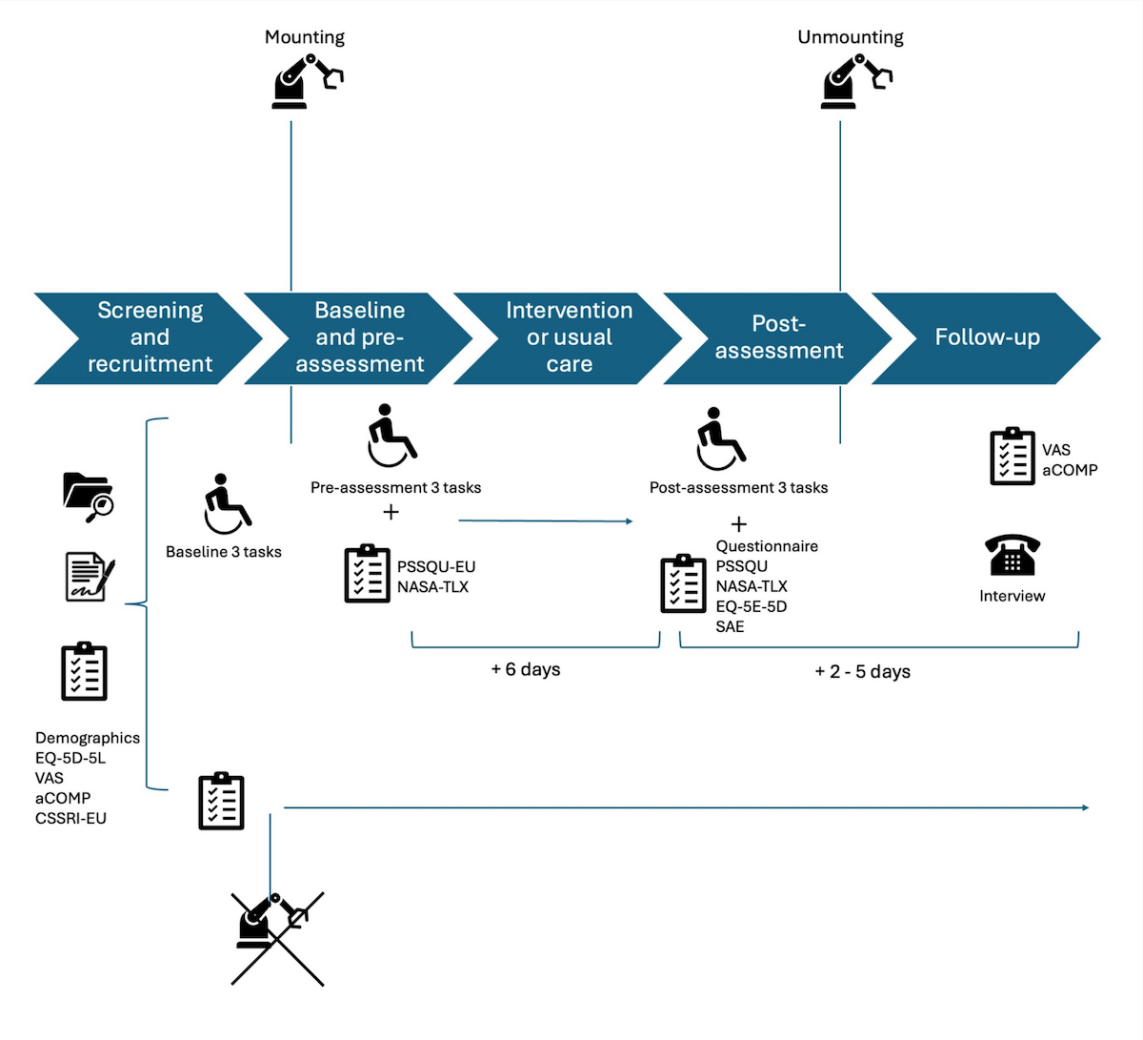
Overview of study procedures. Displayed in the upper row is the intervention group, where screening and recruitment are followed by the baseline assessment, after which the robotic arm system is mounted on the participant’s wheelchair and used to support activities of daily living for 6 days. Within 2 to 5 days postassessments and questionnaires, as well as individual interviews, are conducted. The bottom row shows the nonintervention group, where after screening and recruitment, no clinical testing phase is undertaken but only the completion of the prequestionnaires. aCOPM: Questionnaire on independence in everyday life adapted from Canadian Occupational Performance Measure; CSSRI-EU: Client Socio-Demographic and Service Receipt Inventory-European Version; NASA-TLX National Aeronautics and Space Administration-Task Load Index; PSSUQ: Post-Study System Usability Questionnaire; VAS: Visual Analogue Scale.

The data collected from these participants will also contribute to the health economic evaluation. To enhance the statistical power for detecting potential effects on QoL, an equal number of additional participants, who cannot be allocated to using the robotic arm due to individual reasons for technical incompatibility (eg, wheelchair incompatibility or environmental constraints), will be recruited from the same eligible study population [35]. Recruitment began in April 2025, with an anticipated enrollment period concluding in December 2025. A complete overview of the study procedures can be found in Figure 1.

### Outcomes

Primary and secondary outcomes will be assessed at 3 time points: first, at inclusion and preassessment; second, at the primary endpoint, following 6 days of use with the robotic arm mounted on the wheelchair; and third, at the secondary endpoint, 2 to 3 days after the robotic arm has been unmounted (Table 1).

**Table 1.** Schedule of study procedures and assessments.

Study contacts	Screening and recruitment	Inclusion	Baseline assessments	Preassessment	Intervention or usual care	Primary endpoint	Secondary endpoint
Time	10 days before clinical phase (without robotic arm)	7 days before clinical phase (without robotic arm)	(Without robotic arm)	After 45 minutes training (with robotic arm)	6 consecutive days	After 6 consecutive days (with robotic arm)	2-5 days after clinical phase (without robot arm)
Informed consent form	✓	—^a^	—	—	—	—	—
Informal questionnaire	—	—	—	—	—	✓	—
Demographics^b^	—	✓	—	—	—	—	—
Primary outcome: usability							
Assessment of 3 tasks (pick up, drink, and push a button)	—	—	✓	✓	—	✓	—
PSSUQ^c^	—	—	—	✓	—	✓	—
NASA-TLX^d^	—	—	—	✓	—	✓	—
Secondary outcome: increased QoL^e^ with the use of the robotic arm							
EQ-5D-5L	—	✓	—	—	—	✓	—
Pain with VAS^f^	—	✓	—	—	—	—	✓
aCOPM^g^	—	✓	—	—	—	—	✓
CSSRI-EU^h^	—	✓	—	—	—	—	—
Personal interview	—	—	—	—	—	—	✓

a Not applicable.

b Age; sex; time after disability; height of the lesion; completeness of disability; brand and model of powered wheelchair; spasticity and tonus of upper extremities; mobility of upper extremities; home setting.

c PSSUQ: Post-Study System Usability Questionnaire.

d NASA-TLX: National Aeronautics and Space Administration Task Load Index.

e QoL: quality of life.

f Pain with VAS: pain with Visual Analogue Scale.

g aCOPM: Questionnaire on independence in everyday life, adapted from Canadian Occupational Performance Measure.

h CSSRI-EU: Client Sociodemographic and Service Receipt Inventory.

### Data Management

Quantitative study data from the forthcoming evaluation of the robotic arm and the economic evaluation will be managed using REDCap electronic data capture tools. REDCap is a secure, web-based software platform designed to support data capture for research studies. Password-protected accounts will be created only for authorized study team members, and the level of database access granted to each member depends on their respective roles within the study [36]. Due to the stacked structure of the clinical phase, it will be possible for the research team members to identify the study participants based on the time of data collection; however, further processing of the data takes place anonymously. Daily log files capturing how, when, and where the robotic arm is used, along with data on GUI interactions during ADLs, will be automatically stored in a secure Microsoft Teams folder accessible only to the research team. They will be used to document usage patterns, monitor system performance, and support both technical diagnostics and research evaluation of the WMRA. The log files will contribute to further development and optimization of the system based on the real-world user interaction.

Qualitative data will be saved in a protected file on Microsoft Teams with access only to members of the research group. Data will be pseudonymized with a number, but study participant identification can take place with the time point of data collection. All data, including both electronic and paper-based records, will be retained for an indefinite period. The project leader is responsible for securely storing the research data for at least 10 years following the completion or early termination of the study.

### Data Analysis

Quantitative data will be analyzed using the R statistical computing environment (version 2025.05.0+496; R Core Team) [37], MATLAB (version R2024a; MathWorks) [38] and/or Python (version 3.13; Python Software Foundation) [39]. Descriptive statistics will be used to describe the characteristics of each participant, depending on the nature and distribution of the data, including mean (SD) or median (IQR) for age, disability, and questionnaire data, while categorical variables such as sex will be summarized using frequencies and percentages. The use of standardized instruments such as the EQ-5D-5L will allow for both between- and within-group effects about pre- and postintervention differences. For the assessment of changes in perceived independence in daily activities, a split-plot design will be applied to aCOPM data, enabling both within-subject and between-group comparisons. Repeated measures ANOVA will be used to assess main effects and interaction effects over time. PSSUQ data will be analyzed using ordinal regression to model Likert-scale responses, supplemented by descriptive statistics and mean item scores for usability subscales. NASA-TLX scores will be evaluated using repeated measures ANOVA or nonparametric equivalents to assess cognitive and physical workload changes over time. Task performance (pick-up, drink, and push button) will be analyzed using completion rates and time-to-complete measures, with baseline (without robotic arm) and pre and post (with robotic arm) comparisons performed using paired tests to assess feasibility.

For the health evaluation, quality-adjusted life years (QALYs) will be calculated using the EQ-5D-5L instrument, following the methodology outlined by Sassi [40]. The QALY estimates will be combined with data from the Care-Related Support Index (CRSI) to calculate the Incremental Cost-Effectiveness Ratio (ICER), providing a measure of cost-effectiveness for the intervention. The robustness of ICER results will be examined through both deterministic and probabilistic sensitivity analyses. The latter will use Monte Carlo simulations to account for parameter uncertainty. Given the absence of a binding or explicitly defined ICER threshold in Switzerland, cost-effectiveness acceptability curves (CEACs) will be generated to illustrate the probability that the intervention is cost-effective across a range of willingness-to-pay thresholds. Subgroup analyses will explore heterogeneity in cost-effectiveness.

Qualitative data from informal postquestionnaire responses and semistructured interviews will be analyzed using qualitative content analysis, following an inductive approach to allow themes to emerge from the data. Interviews, transcribed according to predefined rules (Kuckartz and Rädiker [41]), will be analyzed using the AI-based software noScribe [42]. The rapid and rigorous data reduction technique will be used for iterative data reduction, enabling synthesis and thematic comparison with quantitative outcomes [43].

In the final stage of the data interpretation, the quantitative and qualitative results will be integrated in line with the convergent parallel mixed methods design. Findings will be compared across methodological strands to generate a comprehensive understanding of the feasibility, usability, and personal impact of the WMRA. With this, an explanation for possible divergences between performance outcomes (3 tasks, PSSUQ, and NASA-TLX) and user satisfaction, as well as contextual factors influencing variability in quantitative outcomes, can be identified and given. The refinement of the WMRA as a prototype can be established further incorporating relevant user-centered adjustments. In order to minimize the bias of disproportionate emphasis on either type of data, which come along with the different nature of the data types and represent a challenge of mixed methods research [44], regular multidisciplinary analysis meetings will be held. The meetings involve experts in physiotherapy, engineering, health economics, and will ensure a balance of both data types. Furthermore, findings will be cross-validated through triangulation where quantitative and qualitative results will be examined in relation to each other [45].

### Missing Data

To minimize missing data, a stepwise protocol will be implemented to ensure timely and complete data collection at all assessment points. Participants will receive detailed instructions and reminders via REDCap, and research team members will be responsible for verifying questionnaire completion during the study. Despite preventive measures, incomplete data sets will be included in the analysis. If missing values occur, they will be addressed using a multiple imputation approach suitable to the type and extent of the missing data. Recruitment will continue until the predefined sample size is achieved, ensuring adequate statistical power for both usability and health economic analyses.

### Ethical Considerations

A formal clarification of jurisdiction was submitted to the competent cantonal ethics committees to determine whether the study falls under the scope of the Swiss Human Research Act. The committees reviewed the project and declared themselves not responsible, as the study does not fall under the Human Research Act (Art. 2, Abs. 1) [46]. The project does not involve a medical product, biological material, or any modification of standard care. This decision is documented under BASEC-Nr: REq-2022‐01492. Although formal ethics approval was not required, the study will be conducted in accordance with recognized ethical standards, including respect for the dignity, privacy, and autonomy of all participants. All participants provided informed consent prior to participation in the study. Participant data will be pseudonymized and stored securely, with access restricted to authorized study personnel. Participants may withdraw from the study at any time without providing a reason and without any negative consequences. Appropriate safety measures will be implemented throughout the study, including continuous monitoring for adverse events and predefined protocols for incident response, in line with the Declaration of Helsinki [47].

## Results

Recruitment for this study began in April 2025. As outlined in this protocol, up to 15 participants meeting the inclusion criteria will be enrolled in the phase of the robotic arm intervention, with an additional group of up to 15 participants contributing data only for the health economic evaluation. At the time of the manuscript submission, the study is actively recruiting participants, and enrollment and data collection are proceeding according to the predefined study timeline, with no major deviations from the protocol.

As this manuscript reports a study protocol, no outcome data are available at this stage. Upon study completion, results related to feasibility, usability, and the health economic evaluation will be reported.

## Discussion

### Anticipated Findings

This study aims to evaluate the feasibility, usability, and potential health-economic impact of a WMRA, focusing on whether the system is user-friendly and applicable in the daily lives of individuals with tetraplegia in posthospitalization settings.

Based on existing evidence, a reduction in care time for assistance in basic ADLs through the use of the robotic arm may be expected. For instance, the Jaco robotic arm has been shown to save approximately 1.31 hours per day for 31 out of 34 users across 16 standard tasks such as grasping a bottle or pressing a button [12]. Similarly, the iARM system demonstrated an average time saving of 1.25 hours per day [48]. These findings suggest that our robotic system has the potential to significantly reduce the time users depend on external support for basic and instrumental ADLs. From an economic standpoint, the implications of such time savings are considerable. In Switzerland, persons with SCI receive on average 27 hours of care per week [49]. If this care were fully provided for, it would amount to approximately CHF 60,000 (US $78,154.14) per year. Therefore, even partial automation of care could alleviate substantial costs, providing a compelling case for broader implementation and reimbursement of assistive robotic technologies. Importantly, these potential cost savings must be considered in conjunction with the anticipated improvements in QoL. By linking reduced care time to measurable gains in QALYs, the intervention’s overall value can be assessed through cost-effectiveness analysis. Furthermore, our study builds on previous user needs assessments, including the work of Hutmacher et al [28], which emphasized the psychological and physical burden placed on informal caregivers, often over extended periods [6]. By offering relief in routine tasks, the robotic arm may help reduce caregiver strain and promote greater autonomy and self-efficacy among individuals with tetraplegia [7]. This is particularly relevant, as increased autonomy is one of the most frequently reported unmet needs among individuals with high cervical injuries. Through independent task performance and reduced reliance on caregivers, robotic arms may contribute positively to self-perceived independence and mental well-being. Our study is designed to assess these domains using validated instruments such as the EQ-5D-5L and aCOPM, providing quantitative insight into the real-life impact of robotic AT.

### Challenges and Limitations

Despite its promise, the implementation of assistive robotic arms is not without technical and logistical challenges. As the robotic system under investigation is a prototype, hardware malfunctions or software issues could require intervention by technical staff or, in worst-case scenarios, require repair. Such disruptions could significantly hinder the daily use and long-term sustainability of the system in real-world environments. Technical issues might also influence not only feasibility but also secondary outcomes including perceived usability, task performance, cognitive workload, and even potentially responses to the QoL. To account for these technical incidents, they will be logged and considered in the interpretation of feasibility, usability, and economic endpoints.

Participant recruitment presents another challenge, a known barrier in comparable AT studies. Previous projects have struggled to recruit sufficient participants, largely due to the complex logistics of home-based interventions. These often disrupt fragile support systems essential to daily survival, placing additional stress on participants and caregivers. By mixing different inpatient settings forms (inpatient rehabilitation facilities, home settings, nursing homes, or long-term care facilities), the difficulty of participant recruitment is addressed. This approach also allows for the identification of possible integration challenges that are essential for scalable deployment of the WMRA and could be further explored in future studies.

Recruitment is further constrained by the small size of the target population. We address this risk by using a snowball recruitment strategy and diversifying outreach through social media, rehabilitation centers, and caregiver networks. Nonetheless, we remain aware of the limitations this poses for the generalizability of our findings.

The expected heterogeneity within the study sample arising from differences in motor capacity, control interface requirements, and available caregiver support poses another relevant challenge in this study. However, this is a variability inherent to the target user population of the WRMA, and we therefore consider this to ensure ecological validity. Neurological level and ASIA grade will be recorded and included as covariates in the analysis to account for these differences and where subgroup analyses will be conducted where appropriate. Additionally, qualitative data from the user will be used to contextualize the quantitative outcomes, ensuring that differences in independence and support availability are properly interpreted.

Finally, the short intervention period of 6 days limits the ability to capture long-term effects on QoL or sustained behavioral adaptation, which are essential for robust QALY estimation. We are aware that within this period, only immediate psychological impacts of first use can be captured and that it will be used exploratorily in this feasibility study. To measure sustained changes in mental well-being, a longer study period needs to be addressed in future works. Since the WMRA tested is still in the prototype stage and there are currently very few comparable publications evaluating feasibility of a WMRA [11,13,50,51], the test duration of 6 consecutive days was chosen for logistical reasons and to give priority to safety monitoring and minimize user fatigue. Considering future studies such as a pilot clinical trial, the use period could be extended to 2 or 3 weeks to improve the quantitative and qualitative insights gained and measured.

### Conclusion and Impact

This protocol describes a real-world convergent parallel mixed methods feasibility study evaluating a wheelchair-mounted robotic arm designed to support activities of daily living for individuals with tetraplegia across posthospitalization settings in Switzerland. By combining standardized usability and workload measures with task-performance testing, usage logs, qualitative interviews, and a health-economic evaluation, the study is designed to generate complementary evidence on functional feasibility, user experience, and potential cost-effectiveness in everyday contexts.

Pending positive findings, this study may contribute to larger-scale evaluation and long-term clinical trials that assess real-world effectiveness, cost-efficiency, and user benefit of assistive robotic arm systems for persons with tetraplegia. The co-development with end users ensures future iteration and that diverse user needs and preferences of the robotic arm are met, making future versions more user-centered and effective. Ongoing collaboration with engineers and health care professionals could help overcome cost barriers and facilitate broader adoption of the robotic arm.

### Patient and Public Involvement

The development process and design of the robotic arm system was iterative and informed by a previous research cross-sectional study that examined AT needs for individuals with tetraplegia [28]. Importantly, individuals with tetraplegia were actively involved from the outset, contributing their lived experiences and feedback throughout the design and laboratory-based testing phases. A first version of the prototype was showcased at the CYBATHLON 2024 in the Assistance Robot Race, successfully completing 10 daily living tasks, including retrieving a parcel from a mailbox or navigating a touchscreen [52].

## Supplementary material

10.2196/78339 Multimedia Appendix 1 Assessments – 3 Tasks.

10.2196/78339 Multimedia Appendix 2 Semistructured interview guide.

10.2196/78339 Checklist 1 GRAMMS checklist.

## Abbreviations

aCOPM: Questionnaire on independence in everyday life, adapted from Canadian Occupational Performance Measure
ADL: activity of daily living
AIS: ASIA Impairment Scale
ASIA: American Spinal Injury Association
AT: assistive technology
CEACs: cost-effectiveness acceptability curves
CRSI: Care-Related Support Index
CSSRI-EU: Client Socio-Demographic and Service Receipt Inventory-European Version
FRIEND: User Friendly Interface for Disabled People
GRAMMS: Good Reporting of a Mixed Methods Study
GUI: graphical user interface
ICER: Incremental Cost-Effectiveness Ratio
NASA-TLX: National Aeronautics and Space Administration-Task Load Index
PSSUQ: Post-Study System Usability Questionnaire
QALY: quality-adjusted life year
QoL: quality of life
REDCap: Research Electronic Data Capture
SCI: spinal cord injury
VAS: Visual Analogue Scale
WMRA: wheelchair-mounted robotic arm
