# Supplementary figures and images for: Assistive Robotic Arm to Support Activities of Daily Living in Individuals With Tetraplegia: Protocol for a Real-World Convergent Parallel Mixed Methods Feasibility Study
Source: JMIR Res Protoc. 2026 Mar 3;15:e78339. doi: 10.2196/78339 (PMC12978970; doi:10.2196/78339)

# Assessments – 3 Tasks


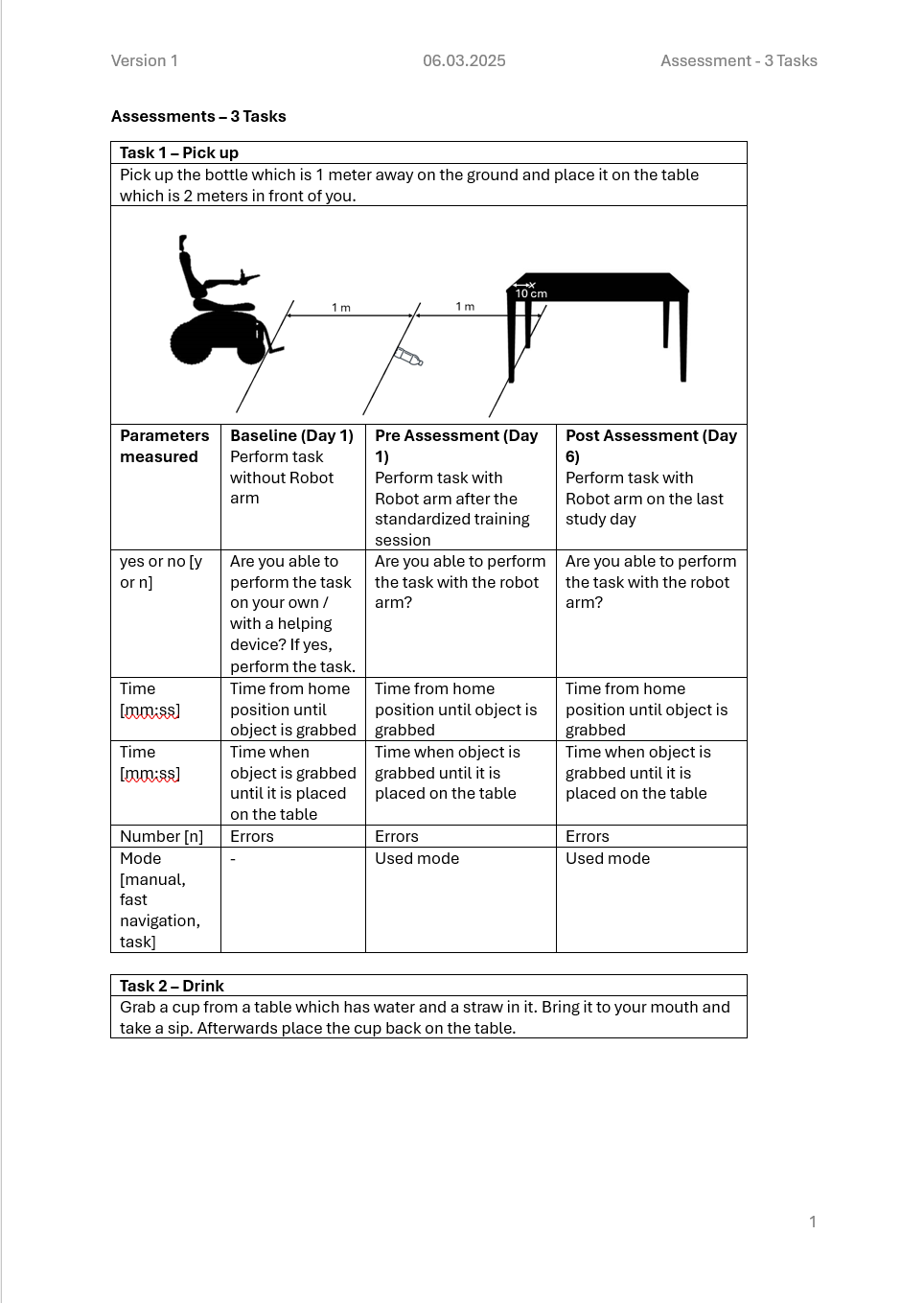


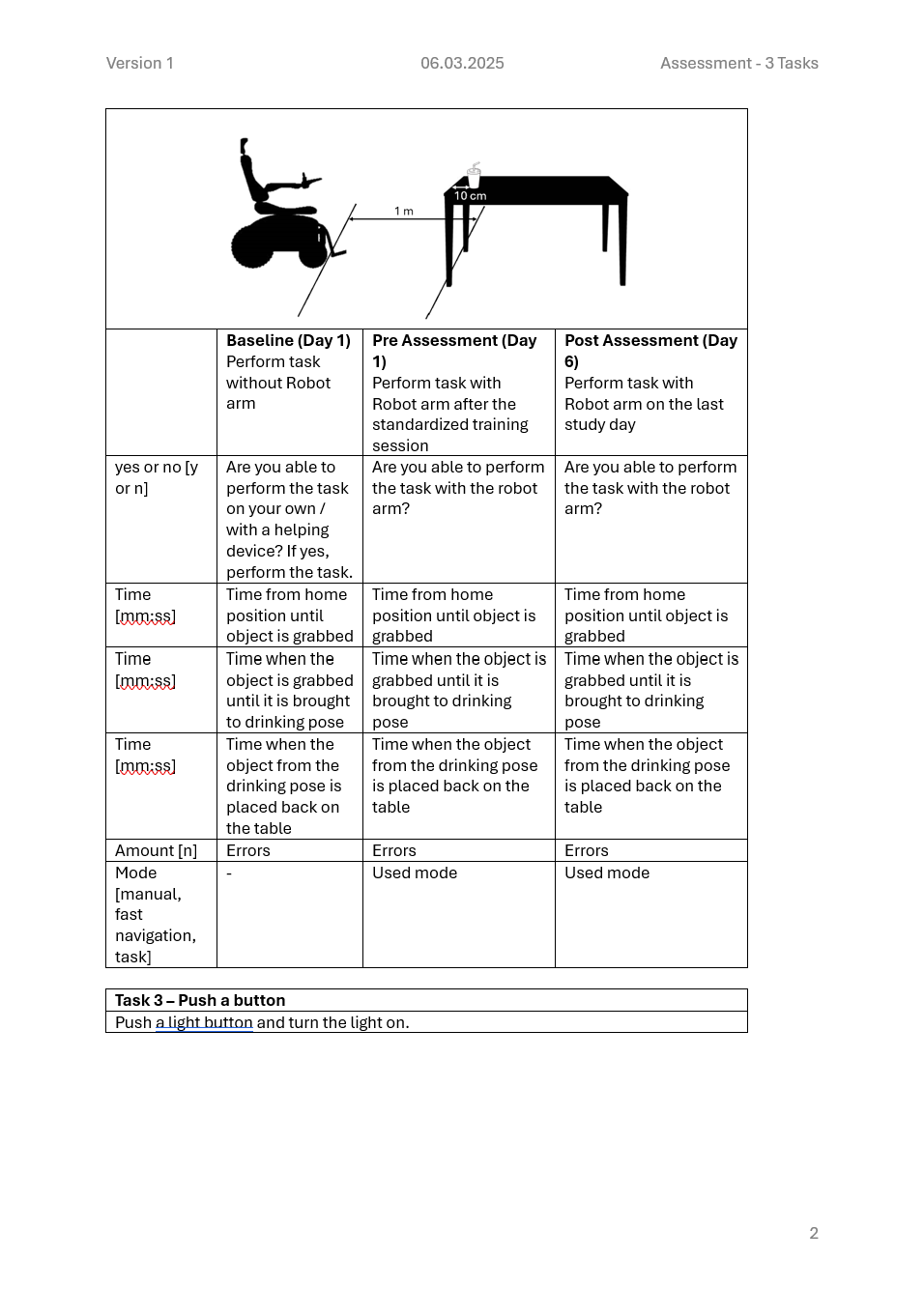


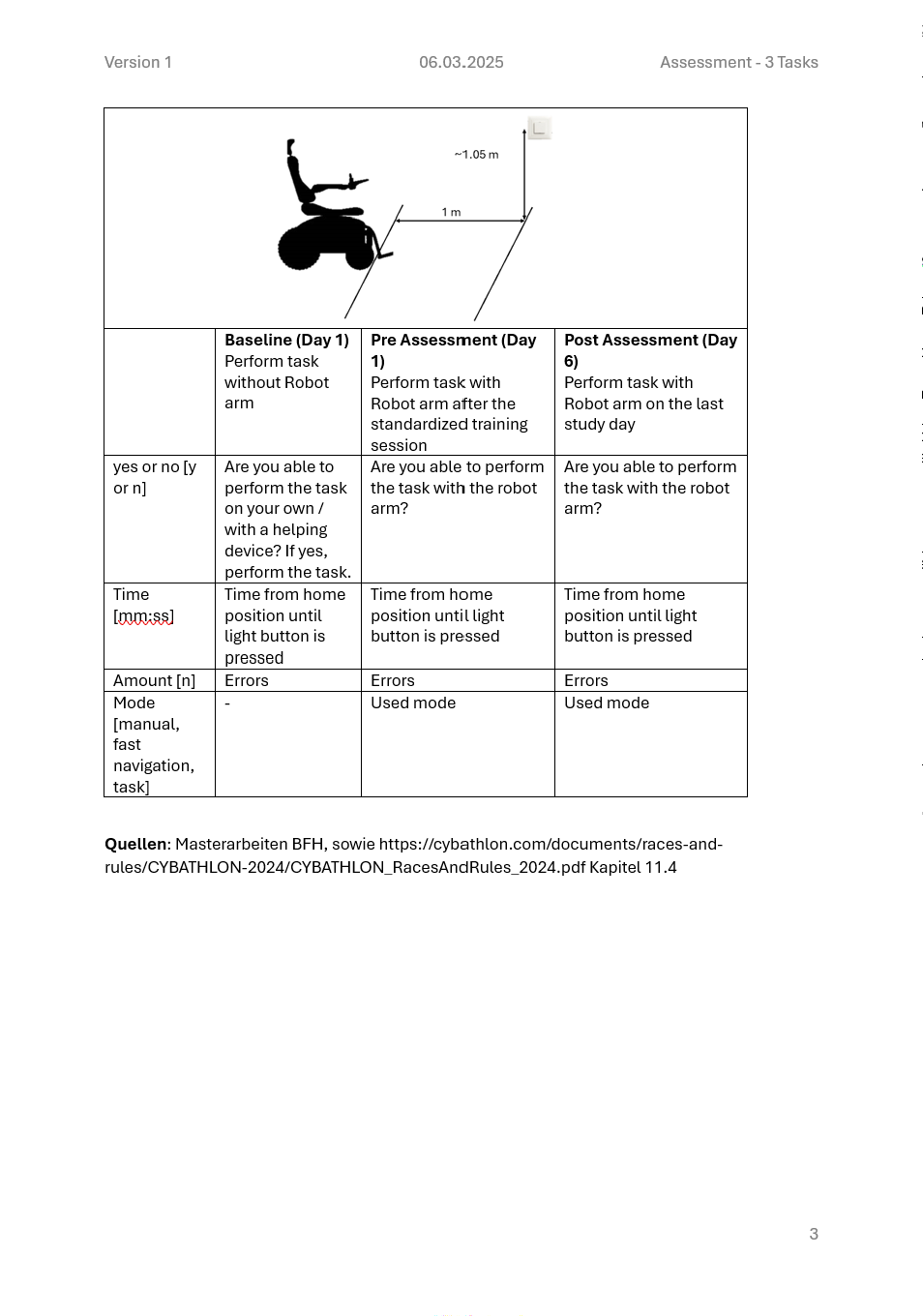

Supplement: Multimedia Appendix 1 [file resprot-v15-e78339-s001.docx]
