# Supplementary material for: Assistive Robotic Arm to Support Activities of Daily Living in Individuals With Tetraplegia: Protocol for a Real-World Convergent Parallel Mixed Methods Feasibility Study
Source: JMIR Res Protoc. 2026 Mar 3;15:e78339. doi: 10.2196/78339 (PMC12978970; doi:10.2196/78339)
Supplement: Multimedia Appendix 2 [file resprot-v15-e78339-s002.docx]

# Semistructured Interview Guide

| **Topics** | **Main Question** | **Specific Follow-up Question** | **Maintenance Question** |
| --- | --- | --- | --- |
| **Introduction** | Could you please tell me your name? |  |  |
| **Physical Complaints** | 1. Has there been any change in your physical complaints through the use of the robotic arm? If yes, what kind? 2. Has there been any change regarding your pain sensation in the upper extremity during the past week? If yes, where and what kind? 3. Have you noticed any fatigue during the use of the robotic arm? | a. Spasticity / muscle tone increase or decrease?  b. Pain increase through the use of the robotic arm or pain reduction?  c. Mobility of the upper extremity? |  |
| **Everyday Activities and Support in Daily Life** | 1. Do you use the robotic arm to support any daily activities? If yes, which ones? 2. What assistive devices have you used to perform everyday activities? 3. Was the robotic arm a useful assistive device for coping with your everyday activities? 4. How has the use of the robotic arm affected your independence? |  |  |
| **Experience with the Robotic Arm** | 1. How did you experience the use of the robotic arm during the past week? 2. In which everyday activities was the use of the robotic arm particularly helpful? 3. Were you able to manage without the help of others through the use of the robotic arm? 4. How did you feel while using the robotic arm? 5. Has the use of the robotic arm affected your self-esteem or self-confidence? 6. Have you received any feedback from friends or family regarding the use of the robotic arm? | a. What advantages have resulted from using the robotic arm?  b. What disadvantages have resulted from using the robotic arm? Were there any moments when it was a hindrance?  c. Can you name other situations in which the robotic arm was helpful or a hindrance? |  |
| **Usage** | 1. What did you like best about using the robotic arm? 2. How and for how long did you use the robotic arm per day (approximately)? 3. How was the use of the robotic arm? What was difficult? What was easy? 4. Can you imagine using the robotic arm in the long term? 5. What long-term advantages or disadvantages do you see in using the robotic arm? 6. Do you feel confident using the robotic arm? 7. Have you used the robotic arm in different environments (e.g., at home, outside, at work)? 8. How did you find the control of the robotic arm? 9. Were there any technical problems or challenges during use? 10. How satisfied were you with the response time of the robotic arm? 11. Were there situations in which the robotic arm did not function properly? 12. Were there sufficient customization options for different tasks? 13. How did you find the user interface of the control software? | 3a. Why was it easy? 3b. Why was it difficult? 4a. If yes, why? 4b. If no, why not? 4c. If you have concerns, what are they?  6a. Were there moments when you felt uncomfortable or uncertain? |  |
| **Questions on User-friendliness** |  |  |  |
| **Development Potential** | 1. How do you see the potential for further development of the robotic arm? | a. What specifically could be improved, or what do you think is most important? | Can you describe how your ideas could look in practice? |
| **Conclusion** | Are there any other aspects that you consider relevant in this context and would like to share? |  |  |
