# Supplementary material for: Assistive Robotic Arm to Support Activities of Daily Living in Individuals With Tetraplegia: Protocol for a Real-World Convergent Parallel Mixed Methods Feasibility Study
Source: JMIR Res Protoc. 2026 Mar 3;15:e78339. doi: 10.2196/78339 (PMC12978970; doi:10.2196/78339)
Supplement: Checklist 1 [file resprot-v15-e78339-s003.pdf]

Multimedia appendix 1: GRAMMS, O'Cathain A, Murphy E, Nicholl J. The quality of mixed methods studies in health services research. J Health Serv Res Policy. 2008;13(2):92-98

Reporting item	Where in Manuscript
(1) Describe the justification for using a mixed methods approach in the research question	Methods: 2.1 Design and setting<br>Mixed methods research design is used to evaluate effectiveness and usability of a WMRA for individuals with tetraplegia. Quantitative analysis explores the feasibility and is used to conduct a health economic analysis, and the qualitative findings allows to help interpret quantitative findings. The use of a mixed methods design allows for a holistic understanding of system performance and user experience.
(2) Describe the design in terms of the purpose, priority and sequence of methods	Methods: 2.1 Design and setting<br>A convergent parallel design with concurrent collection of both quantitative data from standardised and non-standardised questionnaires **and** qualitative insights from individual semi-structured interviews is used. The parallel data collection allows for an alignment and integration if the findings as well as the used of traditional analysis methods of quantitative and qualitative data, relying on familiar, well-established techniques.
(3) Describe each method in terms of sampling, data collection and analysis	Methods and Data Analysis<br>Quantitative:<br>Purposeful sampling<br>Analysis: descriptive statistics, repeated measures ANOVA (or non-parametric equivalents), regression to model Likert-scale<br>Analysis health economic evaluation: QALYs calculation, ICER, CEACs<br>Qualitative:<br>Purposeful sampling<br>Data collection: online/phone semi-structured interviews<br>Analysis: inductive qualitative content analysis
(5) Describe any limitation of one method associated with the present of the other method	Differences in the nature of the data collected pose challenges for the integration. There is a risk of disproportionate emphasis on outcomes that are easier to quantify statistically (e.g. usability scores), potentially overshadowing qualitative insights.
(6) Describe any insights gained from mixing or integrating methods	The integration of quantitative and qualitative measures will allow us to:

	- Explain divergences between performance outcomes and satisfaction<br>- Identify contextual factors that influence variability in quantitative outcomes<br>- Support prototype refinement and prioritize user-centred adjustments
